# Supplementary material for: Designing Inorganic Semiconductors with Cold‐Rolling Processability
Source: Adv Sci (Weinh). 2022 Aug 18;9(30):2203776. doi: 10.1002/advs.202203776 (PMC9596854; doi:10.1002/advs.202203776)
Supplement: Supplementary file 1 — Supporting Information [file ADVS-9-2203776-s001.pdf]

Supporting Information

Designing inorganic semiconductors with cold-rolling processability

Xu-Dong Wang, Jieling Tan, Jian Ouyang, Hangming Zhang, Jiang-Jing Wang, Yuecun Wang, Volker L. Deringer, Jian Zhou, Wei Zhang*, and En Ma*

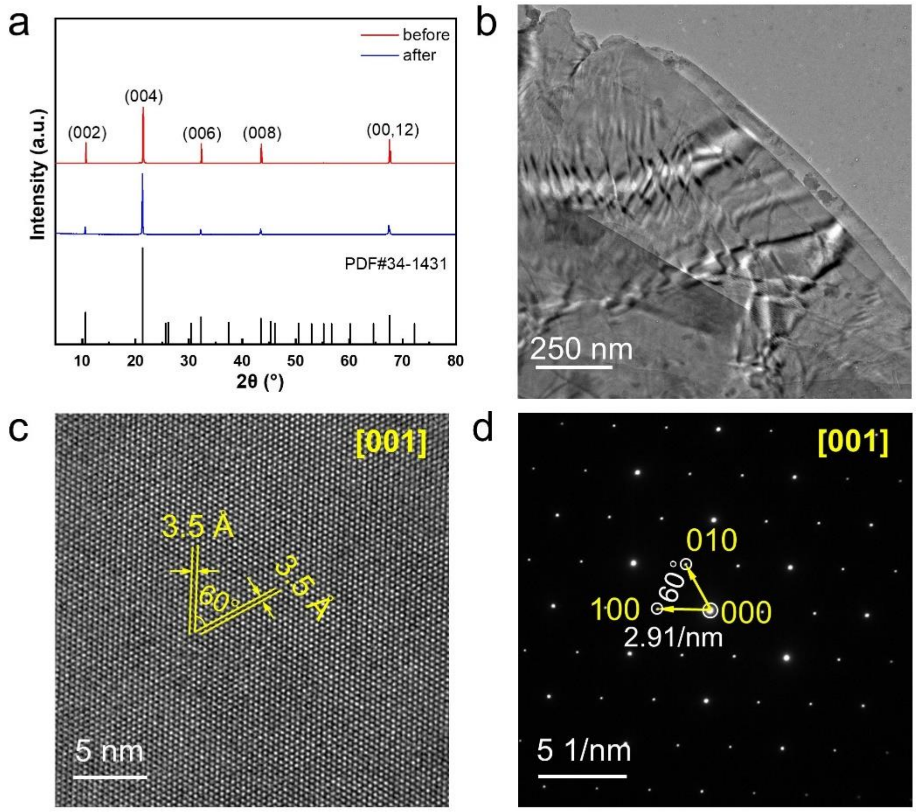

Figure S1. Crystal structure of InSe sample. a X-ray diffraction (XRD) patterns before and after rolling process. **b-c** Bright field TEM images in (b) low and (c) high resolution. **d** Electron diffraction pattern. All these characterizations confirm that our sample is crystalline InSe.

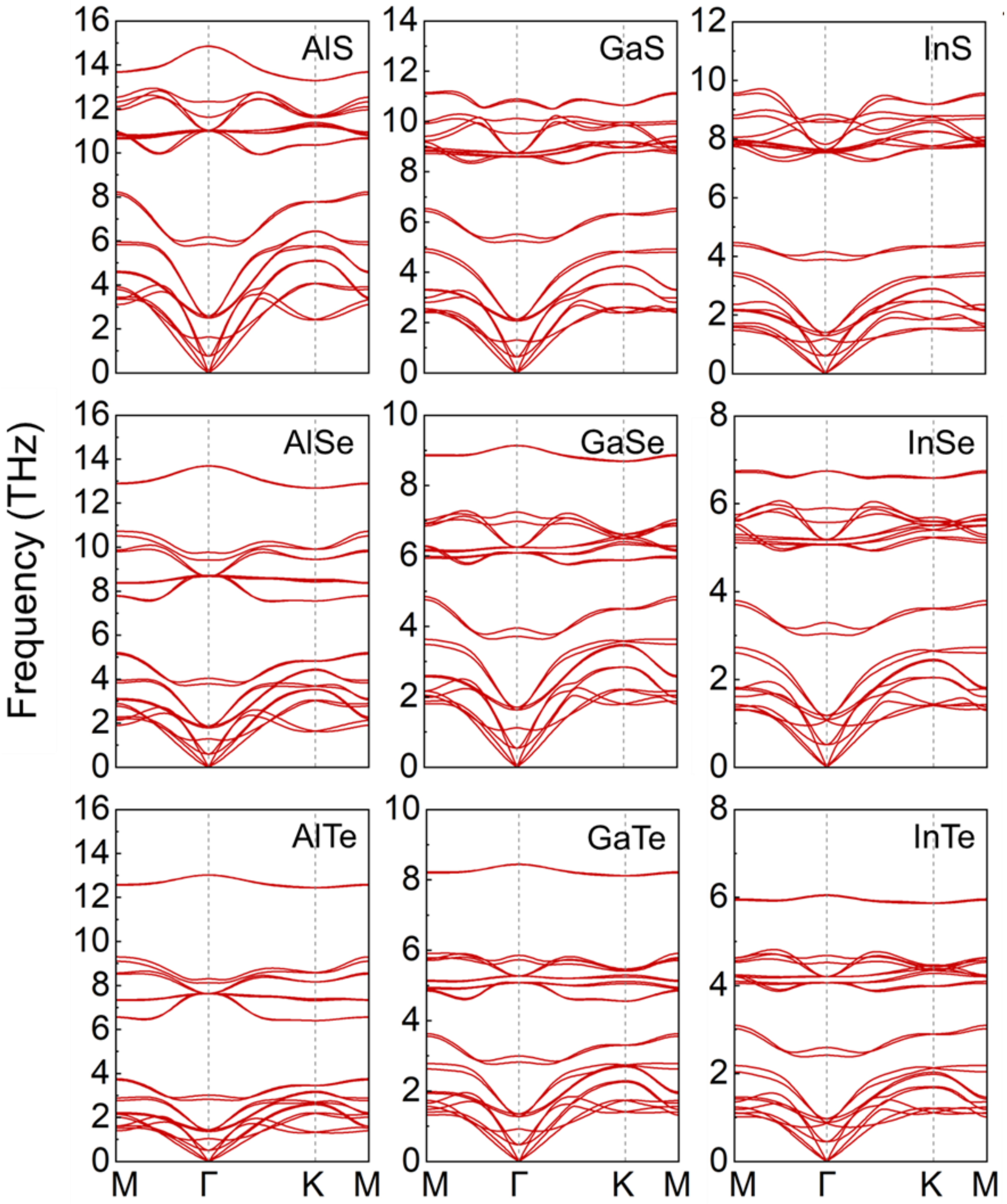

Figure S2. Calculated phonon dispersions for all MX. No imaginary frequency is present, suggesting that all MX in the β-InSe structure are dynamically stable.

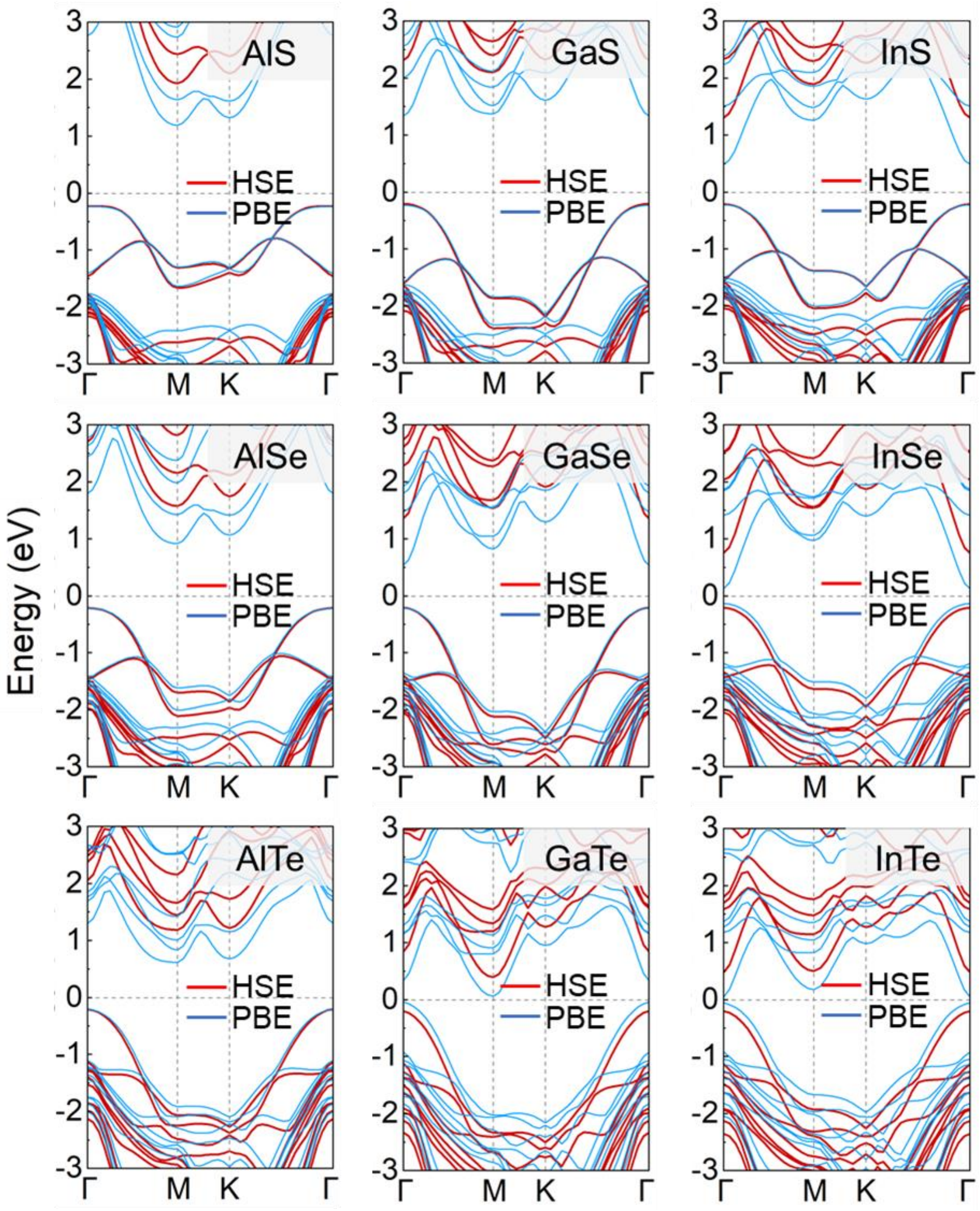

Figure S3. Calculated band structures for all MX. Results based on both PBE and HSE06 functionals are present with considering spin-orbit coupling.

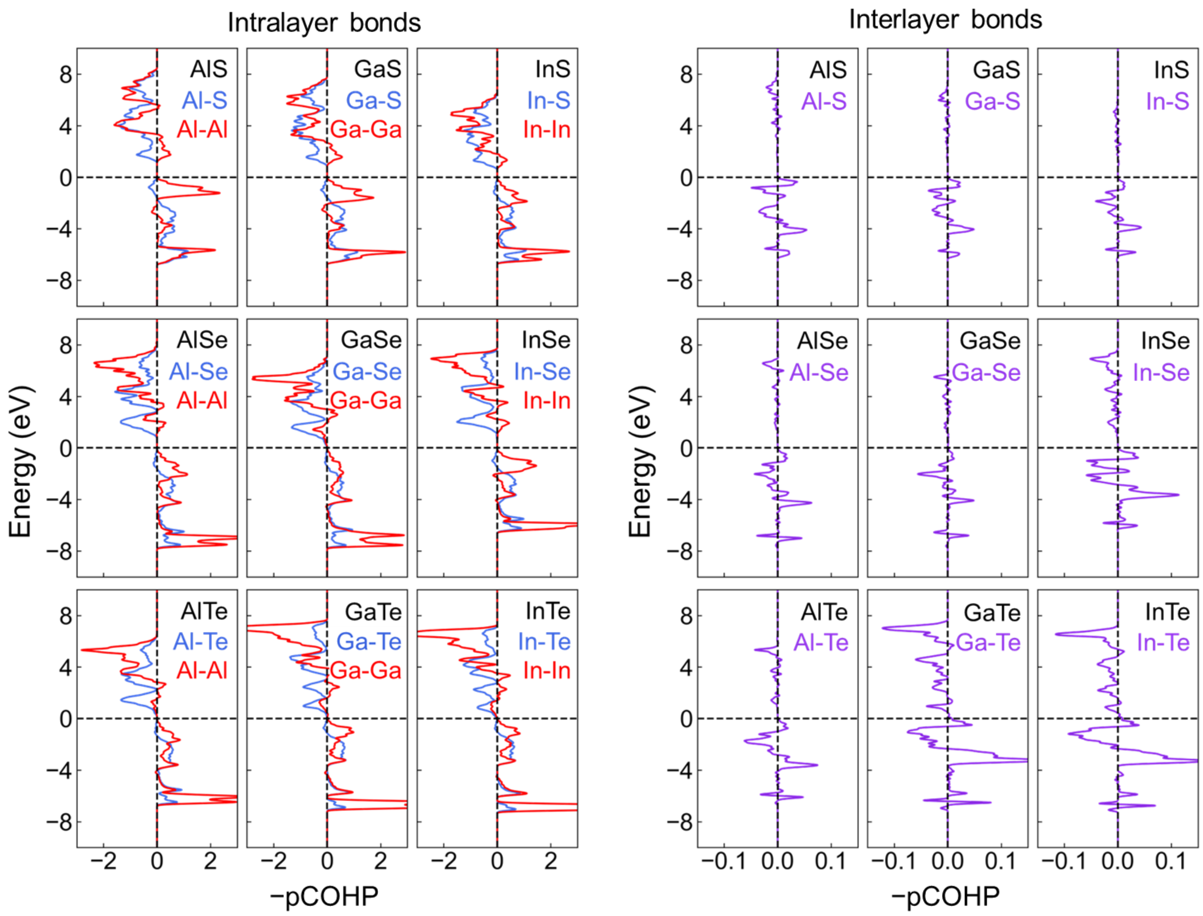

Figure S4. Projected COHP for intra- and interlayer bonds for all MX. The values for intralayer bonds are much larger than those of interlayer bonds, suggesting that intralayer bonds are stronger than interlayer bonds.

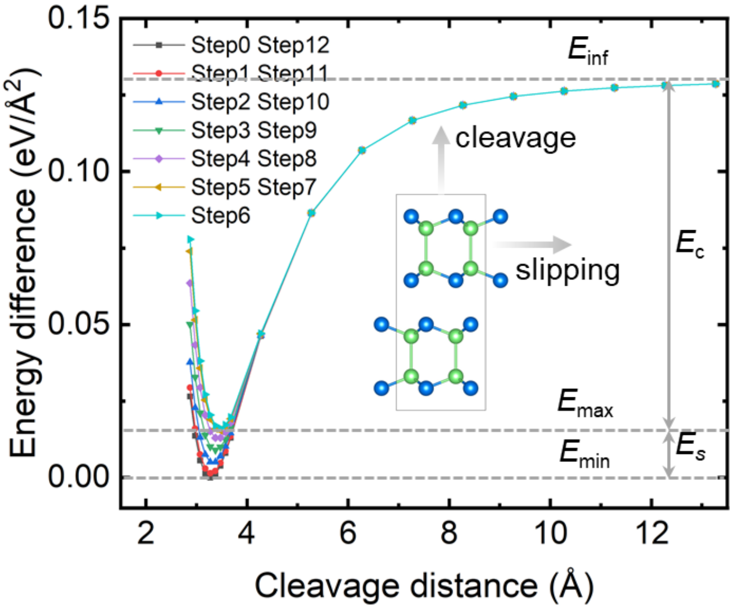

Figure S5. Energy variation with respect to the cleavage distance for each slipping step for GaSe. Slipping energy E_s and cleavage energy E_c are defined as E_{max}–E_{min} and E_{inf}–E_{max}, respectively. E_{min} and E_{max} are the minimum (set as zero) and maximum static energy during slipping, respectively, and E_{inf} is the energy when the layer is fully exfoliated.

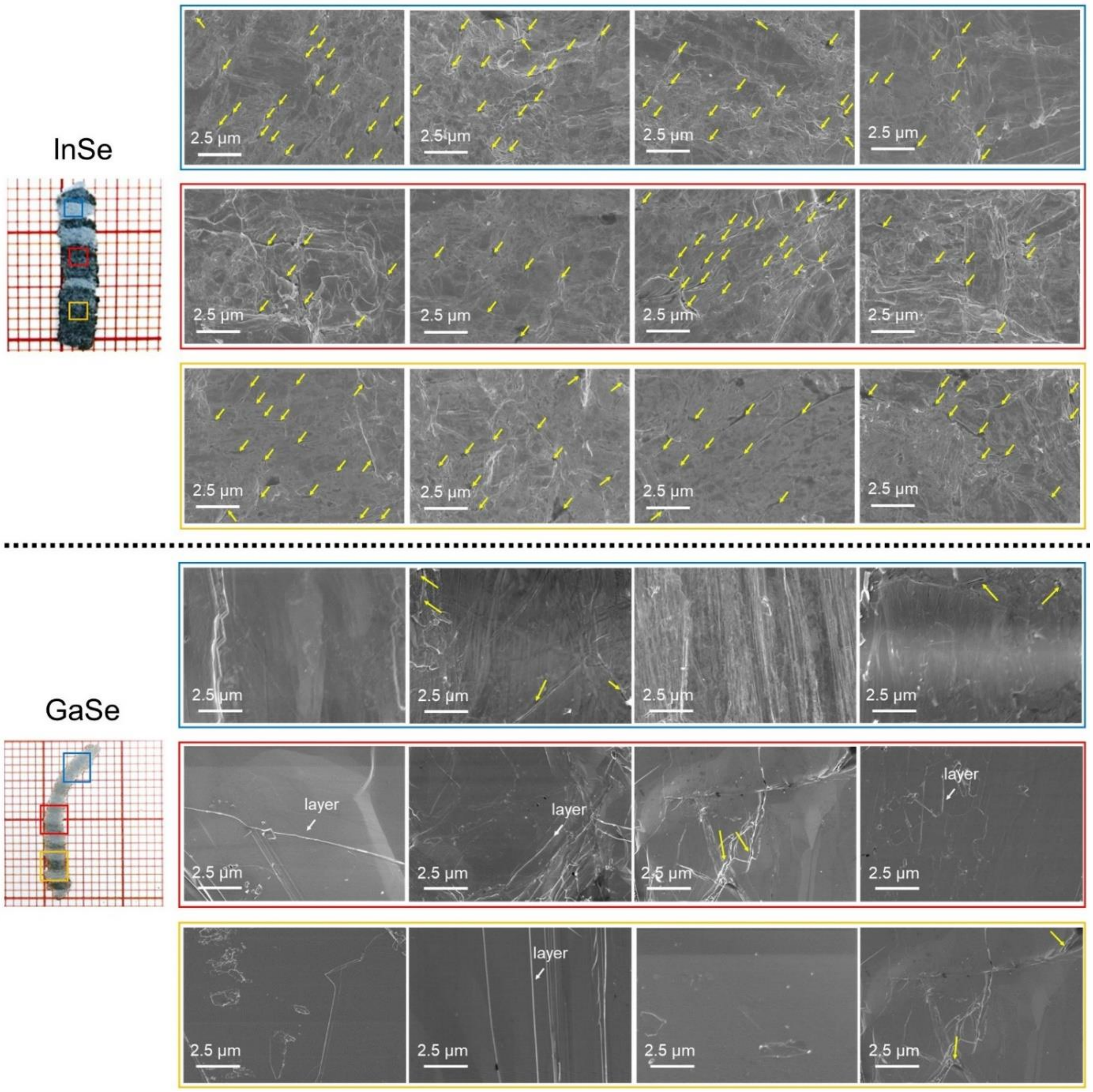

Figure S6. Morphology of roller pressed InSe and GaSe samples. The SEM pictures correspond to three randomly picked regions on the samples which are highlighted as blue, red and yellow boxes. The white and yellow arrows in the SEM pictures point out the edge of layers and the cracks, respectively.

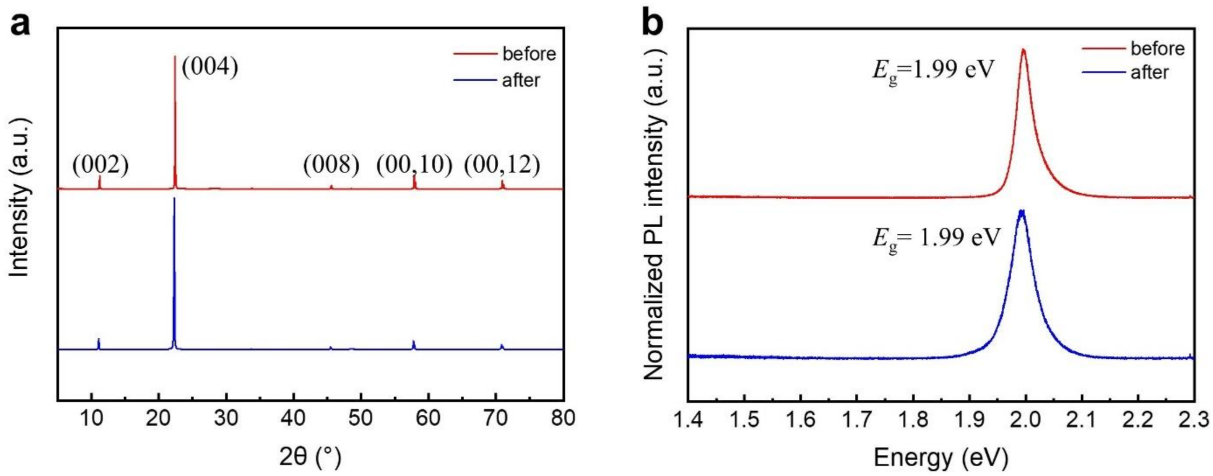

Figure S7. a The measured X-ray diffraction (XRD) and **b** photoluminescence (PL) of GaSe samples before and after roller pressing.

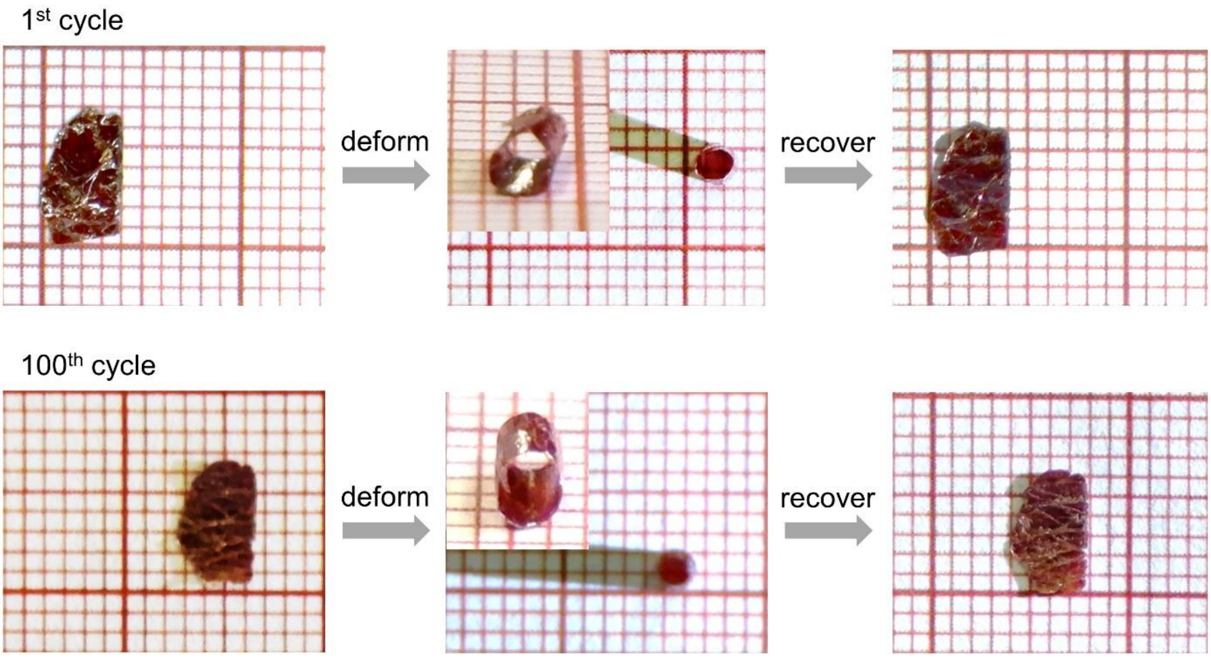

Figure S8. Cyclic deforming tests for a typical GaSe sample. The sample size is 7 × 4 × 0.094 mm^3. Each testing cycle consists of a "deform" and a "recover" stage. The sample can survive without fracturing for more than 100 cycles.
